# Transjugular Intrahepatic Portosystemic Shunt Placement for Portal Hypertension: Meta-Analysis of Safety and Efficacy of 8 mm vs. 10 mm Stents

**DOI:** 10.1155/2020/9149065

**Published:** 2020-10-17

**Authors:** Jiangtao Liu, Eric Paul Wehrenberg-Klee, Emily D. Bethea, Raul N. Uppot, Kei Yamada, Suvranu Ganguli

**Affiliations:** ^1^Department of Radiology, IR Division, Massachusetts General Hospital, Harvard Medical School, Boston, MA 02114, USA; ^2^Department of Gastroenterology, Chinese PLA General Hospital Hainan Hospital, Sanya, Hainan 572016, China; ^3^Liver Center and Gastrointestinal Division, Massachusetts General Hospital, Harvard Medical School, Boston, MA 02114, USA; ^4^Department of Radiology, Division of Interventional Radiology, Boston Medical Center, Boston University School of Medicine, USA

## Abstract

**Introduction:**

Hepatic encephalopathy (HE) following transjugular intrahepatic portosystemic shunt (TIPS) placement remains a leading adverse event. Controversy remains regarding the optimal stent diameter given that smaller stents may decrease the amount of shunted blood and decrease the risk of HE, but stent patency and/or clinical adequacy of portal decompression may also be affected. We aim to provide meta-analysis-based evidence regarding the safety and efficacy of 8 mm vs. 10 mm stents during TIPS placement.

**Methods:**

PubMed, Embase, Cochrane Library, and Web of Science were searched for studies comparing 8 mm and 10 mm stents during TIPS placement for portal hypertension decompression in cirrhotic patients. Randomized controlled trials and cohort studies were prioritized for inclusion. Overall evaluation of quality and bias for each study was performed. The outcomes assessed were the prevalence of HE, rebleeding or failure to control refractory ascites, and overall survival. Subgroup analysis based on TIPS indication was conducted.

**Results:**

Five studies with a total number of 489 cirrhotic patients were identified. The pooled hazard ratio (HR) of post-TIPS HE was significantly lower in patients in the 8 mm stent group than in the 10 mm stent group (HR: 0.68, 95% CI: 0.51~0.92, *p* value < 0.0001). The combined HR of post-TIPS rebleeding/the need for paracentesis was significantly higher in patients in the 8 mm stent group than in the 10 mm stent group (HR: 1.76, 95% CI: 1.22~2.55, *p* value < 0.0001). There was no statistically significant difference in the overall survival between the 8 mm and 10 mm stent groups. The combined risk of HE in the variceal bleeding subgroup was statistically lower (HR: 0.52, CI: 0.34-0.80) with an 8 mm stent compared with a 10 mm stent. The combined risk of both rebleeding/paracentesis and survival was not statistically significant between 8 mm and 10 mm stent use in subgroup analysis.

**Conclusion:**

8 mm stents during TIPS placement are associated with a significant lower risk of HE compared to 10 mm stents (32% decreased risk), as well as a 76% increased risk of rebleeding/paracentesis. Meta-analysis results suggest that there is not one superior stent choice for all clinical scenarios and that the TIPS indication of variceal bleeding or refractory ascites might have different appropriate selection of the shunt diameter.

## 1. Introduction

Transjugular intrahepatic portosystemic shunt (TIPS) placement for portal pressure decompression is a well-established treatment for complications of portal hypertension in cirrhotic patients [1–4]. New or worsened hepatic encephalopathy (HE) is one of the main adverse events after TIPS, with no pharmacological treatment able to completely prevent its incidence [5]. TIPS placement affects hepatic hemodynamics by reducing portal blood inflow to hepatocytes, decreasing hepatic portal perfusion and increasing ischemic injury with decreased hepatic function [6]. The amount of portal blood shunting also prevents hepatic detoxication of the blood and is closely related to post-TIPS HE [7]. The choice of a stent diameter, and therefore the shunt size, balances the demands of portal decompression to prevent portal hypertension complications and shunt-related encephalopathy. Controversy remains regarding the optimal stent diameter owing to the theory that smaller stents may decrease the amount of shunting blood and decrease the risk of HE, but stent patency or clinical adequacy of portal decompression is also affected [8].

In the past decade, 10 mm diameter stents have been used most frequently during TIPS procedures, with reported HE rates of nearly 40% [2, 9]. Underdilation of 10 mm stents at the time of TIPS creation, to 8 mm for example, is a utilized technique to decrease HE incidence, but this technique has not proven to be long-lasting [10–12]. Riggio et al. were the first to compare TIPS placement with 8 mm and 10 mm stents, showing that 8 mm stents lead to significantly less efficient control of portal hypertension with recurrence or persistence of portal hypertension complications in the majority of patients [13]. Another study comparing small-diameter (majority of 8 mm) TIPS with the standard treatment for prevention of variceal rebleeding revealed a significant lower incidence of rebleeding in the 8 mm group, with just a slightly higher prevalence of HE [14]. Other prospective and retrospective studies comparing 8 mm and 10 mm stents in relation to HE, rebleeding, ascites, and survival have shown mixed results in favor of 8 mm or 10 mm stents [15, 16]. Given this controversy, this study is aimed at providing meta-analysis-based evidence regarding the efficacy of 8 mm vs. 10 mm stents during TIPS placement on HE incidence, control of portal hypertension, and overall survival (OS).

## 2. Materials and Methods

### 2.1. Search Method and Selection of Studies

PubMed, Embase, Cochrane Library, and Web of Science were searched for eligible studies from 1988 (the initial year in which metal stent TIPS procedures were performed) to January 2020. The Web of Science search engine was also used for peer-reviewed publications and conference papers or abstracts to ensure full coverage of information to reduce selection bias. The following keywords were included: “transjugular intrahepatic portosystemic shunt”, “TIPS”, “diameter”, “shunt”, “8-mm”, and “10-mm”. The cited references of original studies and reviews were also searched. The following criteria were employed for study selection: (1) study with full text in English; (2) study design: randomized controlled trial (RCT) or retrospective observational study; (3) study participants: cirrhotic patients receiving TIPS for variceal bleeding and/or refractory ascites; (4) study interventions: TIPS with different stent diameters including 8 mm and 10 mm; and (5) at least one of the following outcomes reported: overall survival (OS), number or prevalence of post-TIPS HE, number or rate of post-TIPS rebleeding, number or rate of post-TIPS failure to control ascites or paracentesis, and number or rate of post-TIPS stent dysfunction. Exclusion criteria included the following: (1) noncirrhotic portal hypertension, (2) Budd-Chiari syndrome or hepatic veno-occlusive diseases, and (3) case series studies. This study has been registered at the International Prospective Register of Systematic Reviews (registration number: CRD42020168695).

### 2.2. Outcome Definitions

We acknowledge that the endpoint and adverse event reporting metrics might not be uniform across studies and often include rates or time-to-event results. Given this, the outcomes utilized in this meta-analysis were based on the results of data extraction. The study outcome includes the prevalence of HE or time to HE, the prevalence of rebleeding or the need for paracentesis, time to rebleeding or the need for paracentesis, mortality, or OS. The prevalence of HE was defined as the number of patients who presented with encephalopathy symptoms during follow-up after TIPS. The rebleeding rate was defined as the number of cases who presented with variceal bleeding during follow-up after TIPS. The need for paracentesis was defined as the number of patients with refractory ascites who still required paracentesis during follow-up after TIPS. The rebleeding prevalence and need for paracentesis were combined to create the category of “rebleeding/paracentesis.” OS was defined as the length of time that the patients were still alive after the date of TIPS or to the endpoint of study. Mortality was defined as the number of patients who died from any reason during follow-up after TIPS.

### 2.3. Risk of Bias Assessment

Two investigators (JL and EWK) independently assigned an overall evaluation of quality and bias for each study with the “revised Cochrane risk of bias tool for randomized trials” (RoB 2.0) [17] or the “risk of bias in nonrandomized studies of interventions” (ROBINS-I) for observational cohort studies [18]. The RoB 2.0 tool evaluated the randomization process, deviation from intended interventions, missing outcome data, measurement of outcomes, and selection of reported results with the overall risk-of-bias judgment as “low risk of bias,” “some concerns,” and “high risk of bias.” The overall evaluation with ROBINS-I criteria was “low,” “moderate,” “serious,” “critical,” and “no information” based on the seven domains evaluated. Any differences in evaluation were resolved with a consensus between the two investigators.

### 2.4. Data Extraction

The trial eligibility determination and extraction of data were performed independently by the two investigators. Agreements were made through consensus discussion. Data were extracted with study features and clinical information levels, respectively. Study feature information included the following: study year, study design, sample size and allocation, stent type, mean follow-up time, and bias risk score. Clinical information included the following: treatment group, age, gender, etiology of cirrhosis, history of HE, ascites, Child-Pugh score or class, portosystemic pressure gradient (PSG) before and after TIPS, and indication of TIPS placement. The time-event information in each study was pooled if accessible. The hazard ratio (HR) and its standard error (SE) were pooled directly if they were reported in the publication. Another method for calculation was to use the data available in the report and back-calculate the values with the Mantel-Haenszel method [19]. For outcomes with binary variables, the numbers of observed events were extracted directly or based on the information reported or, if necessary, by contacting the authors for possible data. The risk ratio (RR) was used to evaluate the pooled effect of binary outcomes.

### 2.5. Statistical Analysis

Heterogeneity was assessed by the *I*^2^ index. Data was pooled with a fixed effects model if *I*^2^ ≤ 50%, indicating insignificant heterogeneity. Otherwise, the results of both the fixed effects and random effects models were reported. The visualization of publication bias of the included studies was evaluated using the funnel plot if the sample size was over 10. The *Z*-test was performed to evaluate the significance of the combined HR or RR estimate. Subgroup analysis was conducted based on TIPS indication (variceal bleeding or refractory ascites). A *p* value of 0.05 was set as the threshold for statistical significance. All analyses were performed using the free software R (R Foundation for Statistical Computing, Vienna, Austria) with the “meta” and “dmetar” packages.

## 3. Results

**Figure 1:**
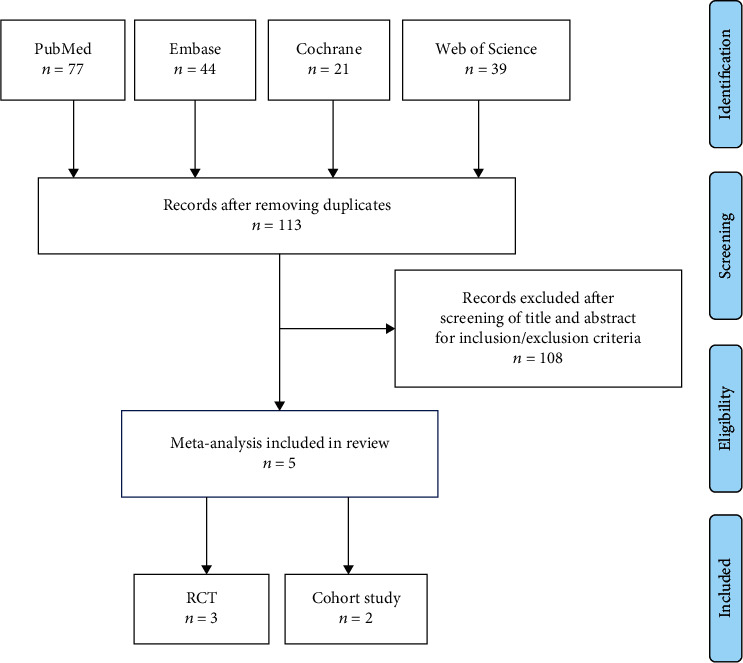
Flow diagram of the meta-analysis study selection process.

Utilizing the described search strategy, we identified a total of 113 publications. 108 of the identified papers were abandoned with the preset inclusion and exclusion criteria. Five studies including 2 RCTs [13, 16] and 3 retrospective cohort studies [15, 20, 21] from 2010 to 2019 were included into the meta-analysis.  Figure 1 provides the flow diagram of publication retrieval, screening, and resulting study selection. Data from Trebicka et al. [20] was retrieved based on a multicenter RCT and propensity score matching for known confounders, so this study was categorized as having an observational feature [20]. The total number of patients reported in the five studies was 489.

### 3.1. Study Characteristics

The five included studies are summarized in Table 1. The two arms for treatment comparisons in all five studies were defined as TIPS placement with 8 mm vs. 10 mm stents. All studies used self-expandable PTFE-covered stents (VIATORR, Gore, Newark, DE, or FLUENCY, Becton Dickinson, East Rutherford, NJ). The indications for TIPS were variceal bleeding in two studies [16, 21], refractory ascites (RA) in one study [15], and both variceal bleeding and RA in two studies [13, 20]. Rebleeding was reported as the probability of remaining free of recurrence and/or persistence of complications due to portal hypertension in one study [13] and as the cumulative incidence of variceal rebleeding in two studies [16, 21]. One study reported the cumulative probability of remaining free from paracentesis for RA [15]. Time-event analysis of HE was reported in four studies [13, 15, 16, 21]. Survival analysis with the log-rank test was reported in three studies [13, 16, 21]. Information on OS was accessed by contacting the authors of [15]. The HR and the corresponding standard error were calculated based on information retrieved in the context of Trebicka et al. [20], where two arms of data were retrieved with the subgroup of 8 mm vs. 10 mm stents (fully dilated plus underdilated). In all the 3 studies with observational features [15, 20, 21], propensity score matching (PSM) was applied to reduce the bias due to confounding variables that could be found in nonrandomized trials. The two RCTs [13, 16] were evaluated with the RoB 2.0 tool, and the three observational cohort studies [15, 20, 21] were evaluated with the ROBINS-I criteria. The bias risk assessment information is summarized in Table 1.

### 3.2. Patient Characteristics

Table 2 summarizes the characteristics of the patients in the five studies. Most of the baseline variables were balanced between the 8 mm and 10 mm groups. Patient age in one study [16] had a slight statistical difference between the two groups (49.4 in 8 mm vs. 52.0 years in 10 mm, *p* < 0.001). In Trebicka et al. [20], the presence of ascites (no/yes; 22/19 in 8 mm vs. 6/35 in 10 mm, *p* < 0.01), Child-Pugh class (A/B/C; 19/18/4 in 8 mm vs. 3/27/11 in 10 mm, *p* < 0.01), and indication for TIPS (bleeding/RA; 29/12 in 8 mm vs. 6/35 in 10 mm, *p* < 0.01) had a statistical difference.

### 3.3. Technical Results

The technical success rate was reported as 100% in all the studies except for Riggio et al. [13], in which an incorrect placement of a stent was subsequently corrected with a second stent. Of all the studies, significant reduction of portal-systemic gradient (PSG) was observed in both the 8 mm and 10 mm stent groups. In Riggio et al. [13], the post-TIPS PSG of the 10 mm group was lower than that of the 8 mm group (6.5 ± 2.7 vs. 8.9 ± 2.7 mmHg, *p* value: 0.0007). Percentages of HE, rebleeding/paracentesis, and mortality were calculated based on the data available in the corresponding studies. The prevalence of post-TIPS HE was between 35.9% and 48.9%, with prevalence of 25%-50% in the 8 mm group and 46.9%-50% in the 10 mm group. The prevalence of rebleeding/paracentesis ranged from 18.1% to 33.3%, with prevalence of 20.3%-54.5% in the 8 mm group and 8.7%-15.5% in the 10 mm group. The mortality rate during follow-up was from 17.8% to 40.2%, with a rate of 20.3%-22.7% in the 8 mm group and 13.0%-27% in the 10 mm group.

### 3.4. Meta-Analysis

**Figure 2:**
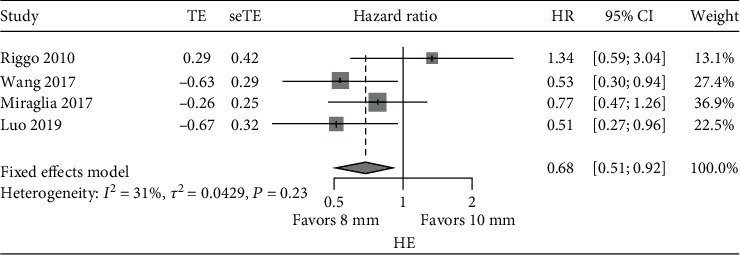
Meta-analysis of HR of HE: 8 mm vs. 10 mm.

According to the heterogeneity analysis, *I*^2^ of both HE and rebleeding/paracentesis was less than 50%. The HR of time to HE or rebleeding/paracentesis amongst the studies was combined with the fixed effects model. The pooled HR of post-TIPS HE was significantly lower in patients in the 8 mm stent group than in the 10 mm stent group (HR: 0.68, 95% CI: 0.51~0.92, *p* value < 0.0001) (Figure 2). The 8 mm stent group had a 32% decreased risk in HE compared to the 10 mm stent group. Compared to the 10 mm stent group, the HR of HE in the 8 mm stent group for four of the studies was between 0.51 and 1.34. Two studies had a statistically significant difference [16, 21], and the other two studies [13, 15] did not show significant differences.

**Figure 3:**
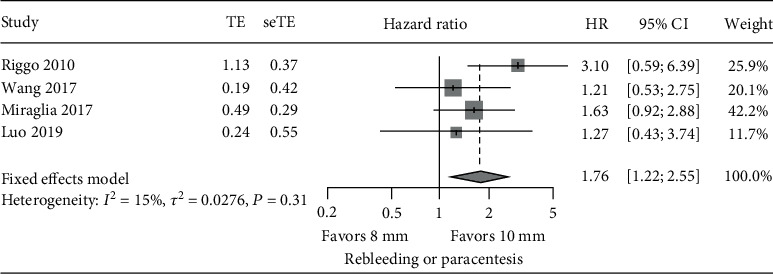
Meta-analysis of HR of rebleeding or paracentesis: 8 mm vs. 10 mm stent TIPS.

The pooled HR of post-TIPS rebleeding/paracentesis was significantly higher in the 8 mm stent compared with the 10 mm stent (HR: 1.76, CI: 1.22~2.55, *p* value < 0.0001), with the 8 mm stent group having a 76% increased risk in rebleeding/paracentesis compared to the 10 mm stent group (Figure 3). Compared with the 10 mm stent group, the HR of rebleeding/paracentesis in the 8 mm stent group was between 1.21 and 3.10, with only Riggio et al. [13] showing a statistically significant difference in favor of the 10 mm group.

**Figure 4:**
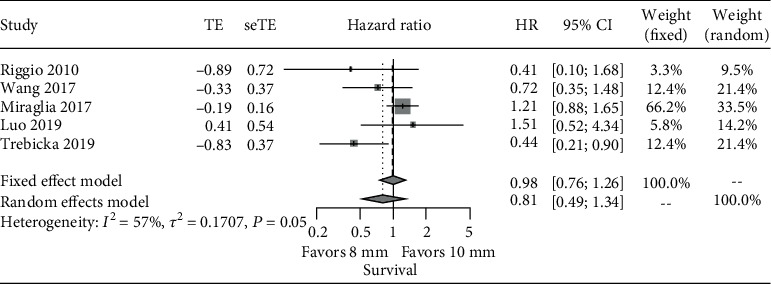
Meta-analysis of HR of survival: 8 mm vs. 10 mm.

*I* ^2^ of the HR for OS was above 50% between studies, so the HR was reported with both fixed and random effects models, and the latter was preferred as the final impression. The pooled HR of OS between the 8 mm and 10 mm stent groups in the included five studies was 0.98 (95% CI: 0.76~1.26, *p* value: 0.859) with the fixed effects model and 0.81 (95% CI: 0.49~1.34, *p* value: 0.411) with the random effects model. There was no statistically significant difference in the risk of death between the 8 mm and 10 mm stent groups (Figure 4). The HR of the 5 studies was between 0.44 and 1.51 with only Trebicka et al. [20] showing a statistically significant difference in survival (HR: 0.44, *p* value: 0.025) in favor of the 8 mm stent group.

**Figure 5:**
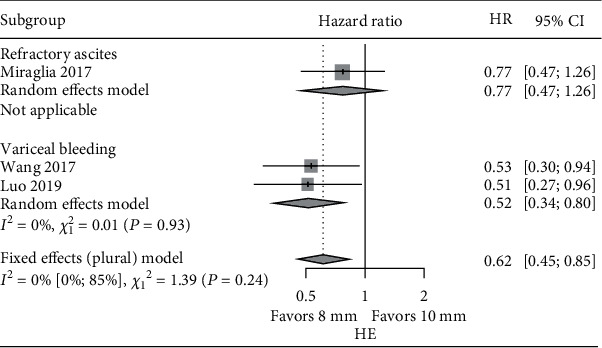
Subgroup meta-analysis of HR of HE in variceal bleeding and refractory ascites: 8 mm vs. 10 mm.

**Figure 6:**
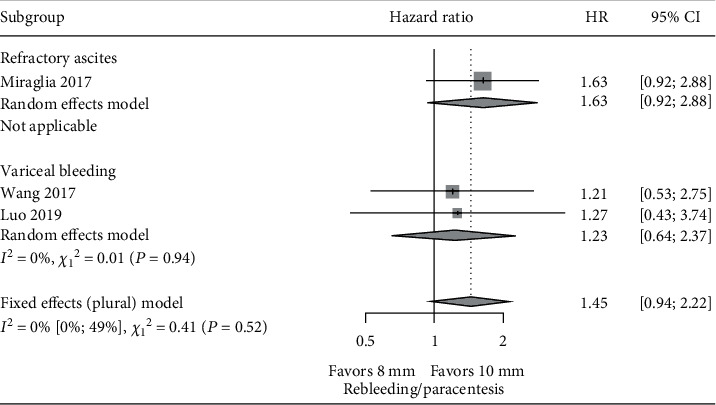
Subgroup meta-analysis of HR of rebleeding or paracentesis in variceal bleeding and refractory ascites groups: 8 mm vs. 10 mm. ^#^The HR of paracentesis was reported in the refractory ascites group. The HR of rebleeding was compared in the subgroup of variceal bleeding.

**Figure 7:**
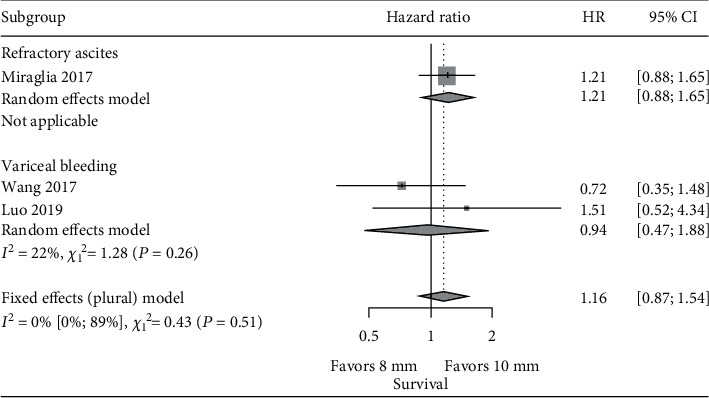
Subgroup meta-analysis of HR of survival in variceal bleeding and refractory ascites groups.

Of the 5 studies included in the meta-analysis, Riggio et al. [13] and Trebicka et al. [20] included both variceal bleeding and refractory ascites, Wang et al. [16] and Luo et al. [21] included only variceal bleeding, and Miraglia et al. [15] focused only on refractory ascites patients. The outcome information corresponding specifically to bleeding or refractory ascites patients is limited. Given this, subgroup analysis was conducted within studies recruiting either variceal bleeding or refractory ascites patients [15, 16, 21]. Results demonstrated that the pooled risk of HE was statistically lower (HR: 0.62, CI: 0.45-0.85) in the 8 mm stent group compared with the 10 mm stent group in the three studies. In the variceal bleeding subgroup, the pooled risk of HE was also statistically lower (HR: 0.52, CI: 0.34-0.80) in the 8 mm stent group compared with the 10 mm stent group. There was only one study with refractory ascites [15]. It did not demonstrate a significant difference of risk of HE between 8 mm and 10 mm stent use (Figure 5). The pooled risk of both rebleeding/paracentesis and survival was not statistically significant between the 8 mm stent and 10 mm stent groups in the subgroup analysis (Figures 6 and 7). The risk of the need for paracentesis with the 8 mm stent group compared to the 10 mm stent group in Miraglia et al. [15] demonstrated marginal significance (HR: 1.63, CI: 0.92-2.88).

## 4. Discussion

The primary result of this meta-analysis shows that the incidence of post-TIPS HE is significantly lower in patients with 8 mm versus 10 mm stents. The 8 mm stent group had a 32% decreased risk of HE compared to the 10 mm stent group. This was in concordance with both Wang et al. and Luo et al. [16, 21], which had statistically significant lower incidences of HE in 8 mm stents, with a HR of 0.53 and 0.51, respectively [16, 21]. Early studies suggested that a stent diameter greater than 12 mm resulted in excessive risk of HE, without additional portal decompression benefits. Further studies established the superiority of 10 mm to 12 mm stents for TIPS procedures in various clinical outcomes, including HE [22]. Meanwhile, a relationship between a smaller shunt diameter and lower incidence of HE has been documented with surgical shunts [23]. In subgroup analysis, the risk of HE in 8 mm stents compared to 10 mm stents remained significant in the variceal bleeding subgroup. Miraglia et al. [15] focused on refractory ascites and did not show a statistical difference between 8 mm and 10 mm stents. To date, there is no definitive statement on the overall superiority of 8 mm versus 10 mm shunts. The challenge in identifying the optimal diameter relates to individual patient characteristics, including the need to balance the necessity of absolute portal pressure reduction against HE risk. What we can report from our present analysis is the superiority of 8 mm stents to 10 mm stents in decreasing post-TIPS HE in portal hypertension-related complications.

Post-TIPS PSG is a critical determinant for the occurrence of HE [24]. In this study, the post-TIPS PSG as well as the extent of decreasing pre-TIPS PSG was comparable between each group in all the recruited studies except for Miraglia et al. [15]. In that study, the post-TIPS PSG was 7.5 ± 2.6 in the 8 mm group vs. 6.5 ± 3.4 mmHg in the 10 mm group (*p* = 0.039). The decrease in PSG was 8.7 mm ± 3.5 mmHg in the 8 mm group vs. 10.4 ± 4.2 mmHg in the 10 mm group (*p* = 0.004). Like most of the recruited studies, previous studies comparing 12 mm and 10 mm stents have not shown a difference in post-TIPS PSG between the two groups [22]. This may be because the subtle decreases in the diameter may not cause remarkable differences in pressure gradient between the portal and hepatic veins. In other words, the pressure gradient might not linearly decrease with an increased shunt diameter after a certain threshold, and the TIPS has reached its maximum effect of decreasing portal pressure. Further increasing the stent diameter may not enhance this effect.

With comparable pressure gradients, a 10 mm stent will receive more portal flow compared to an 8 mm stent, and more unfiltered portal blood will flow directly into the systemic circulation, resulting in an increased risk of HE. In fact, despite the quality of life detriment reported in patients with HE [25], it has been reported as inversely associated with chance of survival [26]. The use of the 8 mm stent in the present analysis leads to decreased incidence of HE. A recent single-arm study [27] of a new controlled expansion stent revealed that most of patients (92%) reached the PSG target (<12 mmHg) with the diameter of 8 mm. With the emerging application of new controlled expansion stents, the choice between 8 mm and 10 mm diameters may be more flexible during TIPS procedures [27, 28] and chosen on a case-by-case basis. However, an 8 mm shunt can be considered when the aim of a PSG of 12 mmHg or a 20% reduction in PSG [29, 30] is satisfactory for clinical indications.

Our study demonstrated a significant difference in risk of rebleeding/paracentesis between the two groups. The 8 mm stent group had a higher risk of rebleeding or the need for subsequent paracentesis. Riggio et al. [13] reported a higher rebleeding rate in patients from the 8 mm stent group, which had a higher post-TIPS PSG than the 10 mm stent patients at the onset of the rebleeding event. Interestingly, the other three studies also reported a trend to higher risk of rebleeding or refractory ascites in the 8 mm stent group with a HR of 1.21-1.63, although without statistical significance. The post-TIPS PSG were similar between both groups, and both were below the recommended threshold of 12 mmHg in the three studies. In Riggio et al. [13], most cases with recurrence and/or persistence of portal hypertension in the 8 mm stent group did not have obvious stenoses on venography, but with an obvious elevated PSG (17.5 ± 5.4 mmHg) compared to immediate TIPS placement. Although the information of PSG was not mentioned in the 10 mm stent group, all cases with recurrence and/or persistence of portal hypertension were shown to have restenosis. The higher rebleeding rate or need for paracentesis of the combined studies in the 8 mm group might not be related directly to the immediate post-TIPS PSG but may represent failure of long-term persistence of decreased portal pressure.

The RCT conducted by Wang et al. [16] demonstrated that TIPS with 8 mm covered stents did not compromise shunt patency compared with 10 mm stents in patients with variceal bleeding. Accordingly, in our subgroup analysis of variceal bleeding indication, the pooled risk of rebleeding did not show a significant difference between 8 mm and 10 mm stents. Miraglia et al., focusing on refractory ascites, did reveal a marginal significance of increased risk of paracentesis requirements in the 8 mm stent group compared with the 10 mm stent group. This suggests that an 8 mm stent does not compromise shunt patency in patients with variceal bleeding but may not be satisfactory for patients with refractory ascites. In fact, the clinical requirements of appropriate post-TIPS PSG may be different between recurrent variceal bleeding and refractory ascites [31, 32] indications, which in turn might have different optimal stent diameters. Although the selection of patients might explain the reason for increased rebleeding or RA incidence in the 8 mm group, it is not definitive.

All-cause mortality is a tangible and clinically relevant outcome. Although different endpoints were reported in the studies, we preferred to combine the time-to-event information between them. The combined HR of OS between the 8 mm stent and 10 mm stent groups was 0.81 and did not reach statistical significance. The heterogeneity of HR for OS within the recruited studies is high. This may be the result of wide confidence intervals in each study, indicating that the pooled result of HR is associated with high uncertainty.

We acknowledge some study limitations. The first is the small sample sizes (5 studies). This might weaken the statistical power of the meta-analysis. Secondly, all three retrospective observational studies have conducted propensity score matching (PSM), by which most of the known baseline characteristics in the studies were matched between groups and balanced. But unlike RCT, it may not eliminate the potential bias that arises from any unknown confounders. Due to their study designs, the risk of bias remains moderate to severe in the three studies. A third limitation is the subgroup analysis, which was conducted with only 3 studies recruiting either variceal bleeding or refractory ascites due to specific outcome information inaccessibility. This weakens the persuasive power of the results. Fourth, all the retrieved studies used covered stents, which limits the generalizability of the conclusion. Although bare stents are used much less for TIPS in the era of covered stents, this should be noted because the difference between covered and bare stents is popularly regarded as significant [33]. Lastly, post-TIPS HE is often associated with multiple factors including age, prior HE, and liver function [34]. The shunt diameter should only be included into consideration amongst other important factors that influence the post-TIPS HE.

In conclusion, this meta-analysis demonstrated that 8 mm stents during TIPS placement are associated with a significantly lower risk of HE, but a higher risk of rebleeding and/or uncontrolled refractory ascites when compared to 10 mm stents. The OS between 8 mm and 10 mm stent patients is similar. Based on the limited information in the present analysis, we deduce conservatively that the indication of TIPS may indicate specific selection of the shunt diameter, with variceal bleeding being prone to 8 mm stent placement and refractory ascites to 10 mm stent placement. Furthermore, well-designed clinical trials with subgroup TIPS indications should be encouraged to further reveal the optimal choice of 8 mm or 10 mm stents in clinical practice.

## Figures and Tables

**Table 1:** Study characteristics.

Reference	Year	Study design	Sample size (8 mm/10 mm)	Stent type (PTFE-covered)	Mean follow-up time in months (8 mm/10 mm)	Bias risk evaluation^∗∗^
Riggio et al. [13]	2010	Randomized control trial	22/23	VIATORR, Gore	12/15.7	Some concerns
Miraglia et al. [15]	2017	Retrospective cohort study	111/60	VIATORR, Gore	71.7/74.8	Moderate risk
Wang et al. [16]	2017	Randomized control trial	64/63	FLUENCY, Bard	26.9^∗^	Low risk
Trebicka et al. [20]	2019	Retrospective cohort study^#^	41/41	VIATORR, Gore	NA	Serious risk
Luo et al. [21]	2019	Retrospective cohort study	32/32	FLUENCY, Bard	38.7/22.5	Moderate risk

^#^Subgroup cohort data within a randomized controlled trial. ^∗^Reported with overall follow-up time. ^∗∗^RCTs were evaluated with RoB 2.0; cohort studies were evaluated with ROBINS-I.

**Table 2:** Patient characteristics.

Reference	Treatment group	Age (years)	Gender (male/female)	Etiology (viral/nonviral)	History of HE (yes/no)	Ascites (yes/no)	Child-Pugh class (A/B/C)	PSG baseline (mmHg)	Post-TIPS PSG (mmHg)	TIPS indication (bleeding/RA)
Riggio et al. [13]	8 mm	53.1 ± 11.3	15/7	13/9^#^	6/16	15/7	5/10/7	21.3 ± 4.9	8.9 ± 2.7^∗^	12/10
	10 mm	57.1 ± 9.9	13/10	14/9^#^	3/20	18/5	5/15/3	22.1 ± 7.1	6.5 ± 2.7^∗^	9/14
Miraglia et al. [15]	8 mm	58.6 ± 10.6	76/35	63/51	36/75	111/0	0/71/40	16.1 ± 3.7	7.5 ± 2.6	0/111
	10 mm	59.0 ± 10.0	36/24	40/20	20/40	60/0	0/35/25	17.0 ± 4.2	6.5 ± 3.4	0/60
Wang et al. [16]	8 mm	49.4 ± 11.0^∗^	41/23	54/10	NA	32/32	36/25/3	26.2 ± 4.3	8.2 ± 3.0	64/0
	10 mm	52.0 ± 9.7^∗^	37/26	47/16	NA	35/28	35/25/3	24.9 ± 4.3	7.4 ± 3.0	63/0
Trebicka et al. [20]	8 mm	56 (33~81)^∗∗^	29/12	25/16	11/30	19/22^∗^	19/18/4^∗^	NA	NA	29/12^∗^
	10 mm	56 (41~71)^∗∗^	29/12	31/10	14/27	35/6^∗^	3/27/11^∗^	NA	NA	6/35^∗^
Luo et al. [21]	8 mm	52 ± 12	20/12	25/7	0/32	21/11	10/18/4	23.9 ± 6.3	9.2 ± 3.5	32/0
	10 mm	51 ± 11	20/12	23/9	0/32	21/11	12/16/4	24.6 ± 7.3	7.4 ± 3.7	32/0

^#^Reported as alcoholic/nonalcoholic. ^∗^Variables of 8 mm vs. 10 mm groups with significant difference. ^∗∗^Expressed as median (range).
